# Detection of Serratia marcescens in neonatal intensive care units requires a rapid and comprehensive infection control response starting with the very first case

**DOI:** 10.3205/dgkh000383

**Published:** 2021-03-15

**Authors:** Carolin Böhne, Patrick Chhatwal, Corinna Peter, Ella Ebadi, Gesine Hansen, Dirk Schlüter, Franz-Christoph Bange, Bettina Bohnhorst, Claas Baier

**Affiliations:** 1 Department of Pediatric Pulmonology, Allergology and Neonatology, Hannover Medical School, Hannover, Germany; 2 Institute for Medical Microbiology and Hospital Epidemiology, Hannover Medical School, Hannover, Germany

**Keywords:** preterm infants, neonatal intensive care unit, cluster, infection control, outbreak, Serratia marcescens

## Abstract

**Background:** *Serratia* *marcescens* is a well-known and challenging pathogen in neonatal intensive care units. It is responsible for severe infections and can cause nosocomial outbreaks.

**Methods:** We present the infection control response to a *Serratia* *marcescens* cluster which occurred in a tertiary neonatal intensive care unit.

**Results and conclusions**: The presented comprehensive and decisive hygiene management response starting with the very first case aims especially at early detection and immediate interruption of nosocomial transmission. Frequent and sensitive microbiological screening, rigorous spatial isolation of colonized infants, and reinforcing adherence to hand hygiene are essential in this response, which comprises eight measures. It prevented a full-blown outbreak.

## Zusammenfassung

**Hintergrund:** *Serratia* *marcescens* ist ein wohlbekannter, herausfordernder Erreger in der neonatologischen Intensivmedizin. Er kann schwere Infektionen bei Frühgeborenen und nosokomiale Ausbrüche verursachen.

**Methoden:** In der vorliegenden Arbeit präsentieren wir das Hygienemanagement einer *Serratia marcescens* Häufung auf einer tertiären neonatologischen Intensivstation.

**Ergebnis und Schlussfolgerungen**: Das vorgestellte Hygienemanagement, das bereits mit dem ersten auftretenden *Serratia marcescens* Fall angewendet wird, zielt insbesondere auf die frühe Detektion und schnelle Unterbrechung von nosokomialen Transmissionsketten ab. Ein engmaschiges, sensitives mikrobiologisches Screening, eine strikte räumliche Isolation und die Stärkung der Händehygiene-Adhärenz sind zentrale Maßnahmen in diesem Konzept, welches insgesamt 8 Komponenten beinhaltet. Das Konzept war in der Lage, die Häufungssituation rasch zu kontrollieren und zu begrenzen.

## Background

*Serratia marcescens* (Sm) is associated with nosocomial outbreaks in neonatal intensive care units (NICUs) [1], [2], [3], [4], [5]. Preterm infants bear a particular risk for severe infections caused by Sm due to their immature immune system. Besides bloodstream infections (BSIs), Sm can also cause meningitis/brain abscess or conjunctivitis [6]. Overall, the occurrence of Sm in an NICU requires a fast and decisive response to prevent or contain nosocomial spread. Here, we present and discuss a comprehensive infection control response, which was successfully implemented in a tertiary NICU to rapidly control a Sm cluster. 

## Methods

The cluster occurred in an NICU of a German university hospital in May/June 2020. The NICU had 10 beds in three two-bed rooms and one four-bed room. The regular nurse-to-patient ratio was 1:1 to 1:3. Assigned cleaning and healthcare staff serviced the ward. The ward was disinfected twice daily. The incubators were changed weekly and disinfected at a central facility.

According to the recommendations of the Commission for Hospital Hygiene and Infection Prevention at the German Robert Koch Institute, preterm infants were regularly screened for colonization of various bacteria, including Gram-negative rods, during their stay at the NICU. The screening included rectal and respiratory specimens and was performed upon admission and once a week afterwards (every Monday). Sm isolates were molecularly characterized by pulsed-field gel electrophoresis (PFGE) according to an in-house protocol (restriction enzyme SpeI) in a hygiene laboratory that is accredited according to ISO/IEC 17025 by the German national accreditation body. PFGE patterns were compared visually. The Sm cluster was investigated in line with the ORION statement. The institution’s ethics committee approved the analysis (number 9264_BO_K_2020).

## Results

The cluster affected four preterm infants (two pairs of twins). They had a birth weight of 1,150 g and 1,350 g (30+2 weeks) and 1,450 g and 1,590 g (32+1 weeks). All the children tested positive within one week and Sm acquisition was assumed to be nosocomial. Each pair of twins shared one room. All infants were colonized rectally, including three who additionally had positive respiratory specimens. The isolates shared an identical phenotypic antimicrobial susceptibility pattern (susceptible to third-generation cephalosporins, fluoroquinolones and carbapenems). 

**Figure 1:**
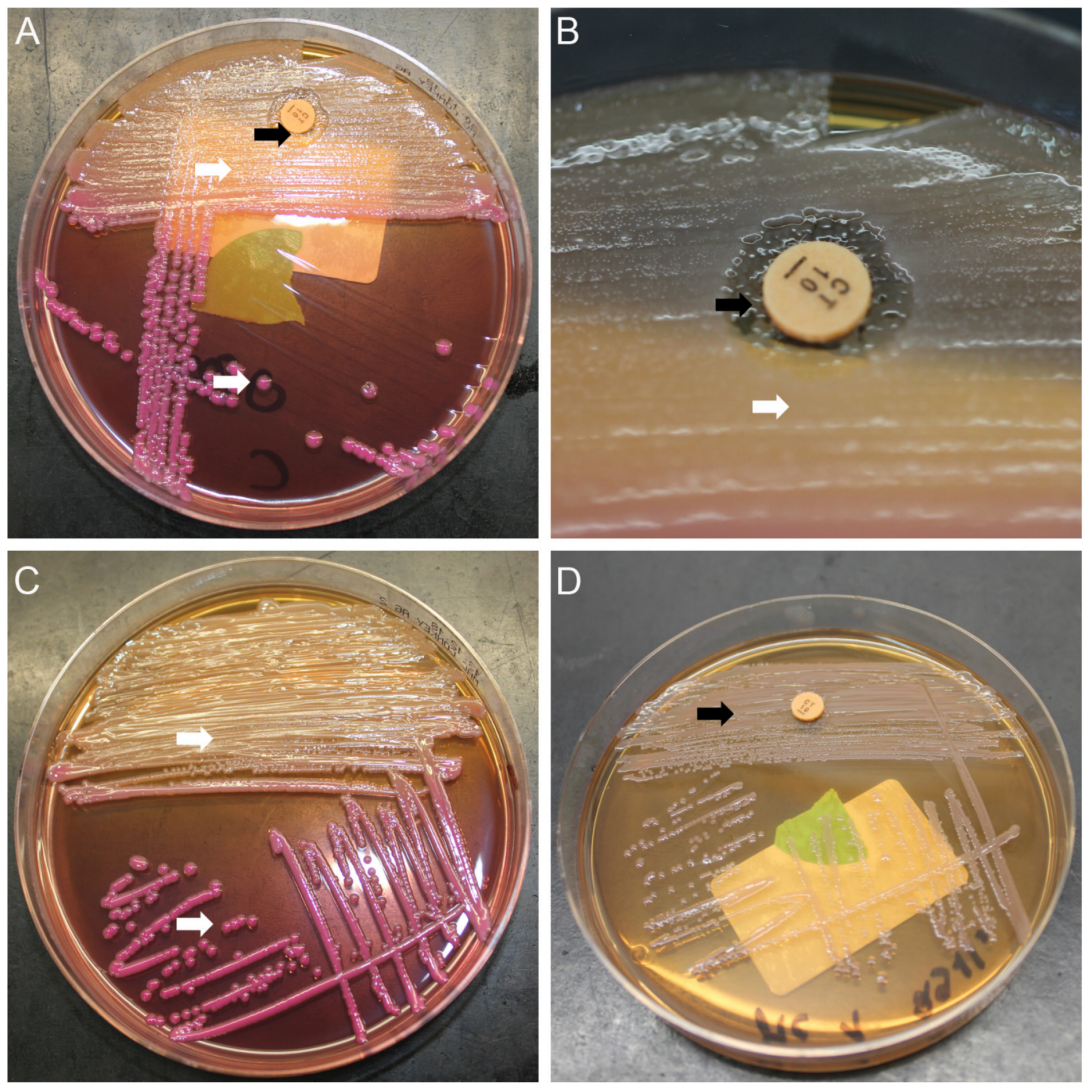
Serratia marcescens microbiological screening A: MacConkey agar with a colistin disk showing the growth of *Klebsiella oxytoca* (white arrows) and *Serratia* *marcescens* (black arrows); *Serratia marcescens* colonies are only visible within the colistin inhibition zone; B: Colistin inhibition zone with *Serratia marcescens* colonies in detail; C: Example of MacConkey agar without a colistin disk; *Serratia marcescens* colonies are masked by the more abundant species (*Klebsiella oxytoca*); D: Pure culture of *Serratia marcescens*, which is not inhibited by colistin due to intrinsic resistance.

The first patient with Sm was detected by routine weekly colonization screening in the NICU. After Sm detection in this first patient, the following measures were implemented immediately:

1. Frequent screening targeting Sm. Another weekly screening day (Thursday) was therefore established. These screening specimens were processed on standard MacConkey agar with a colistin disk to increase sensitivity for Sm. Figure 1 (Fig. 1) shows this approach.
2. Establishment of an interdisciplinary task force to ensure proper and timely information to all stakeholders (neonatologists, infection control staff, microbiologists).
3. Strict spatial isolation (single room or cohort) and contact precautions (gloves, gown). Moreover, the nursing staff were exclusively assigned and did not care for other infants (as far as possible).
4. Discharge or transfer of Sm patients as soon as possible.
5. Reinforcement of adherence to hand hygiene by staff (including external personnel) and parents by frequent on-site training.
6. Clinical alertness towards the development of Sm infection in colonized patients.
7. Molecular characterization to i) verify (or falsify), ii) better understand, and iii) visualize potential transmission.
8. Environmental source search (e.g., the microbiologic examination of inanimate surfaces and medical equipment with contact plates and swabs or microbiologic examination of disinfectant solution and nutrition, including breastmilk).

Sm colonization of the three other patients was detected within one week following the index case. Fortunately, one pair of twins was successfully transferred from the NICU within one week of Sm detection. The other pair of twins, however, stayed in the NICU for a total of 43 days. Sm was not detected in this pair of twins by the end of their hospital stay. 

**Figure 2:**
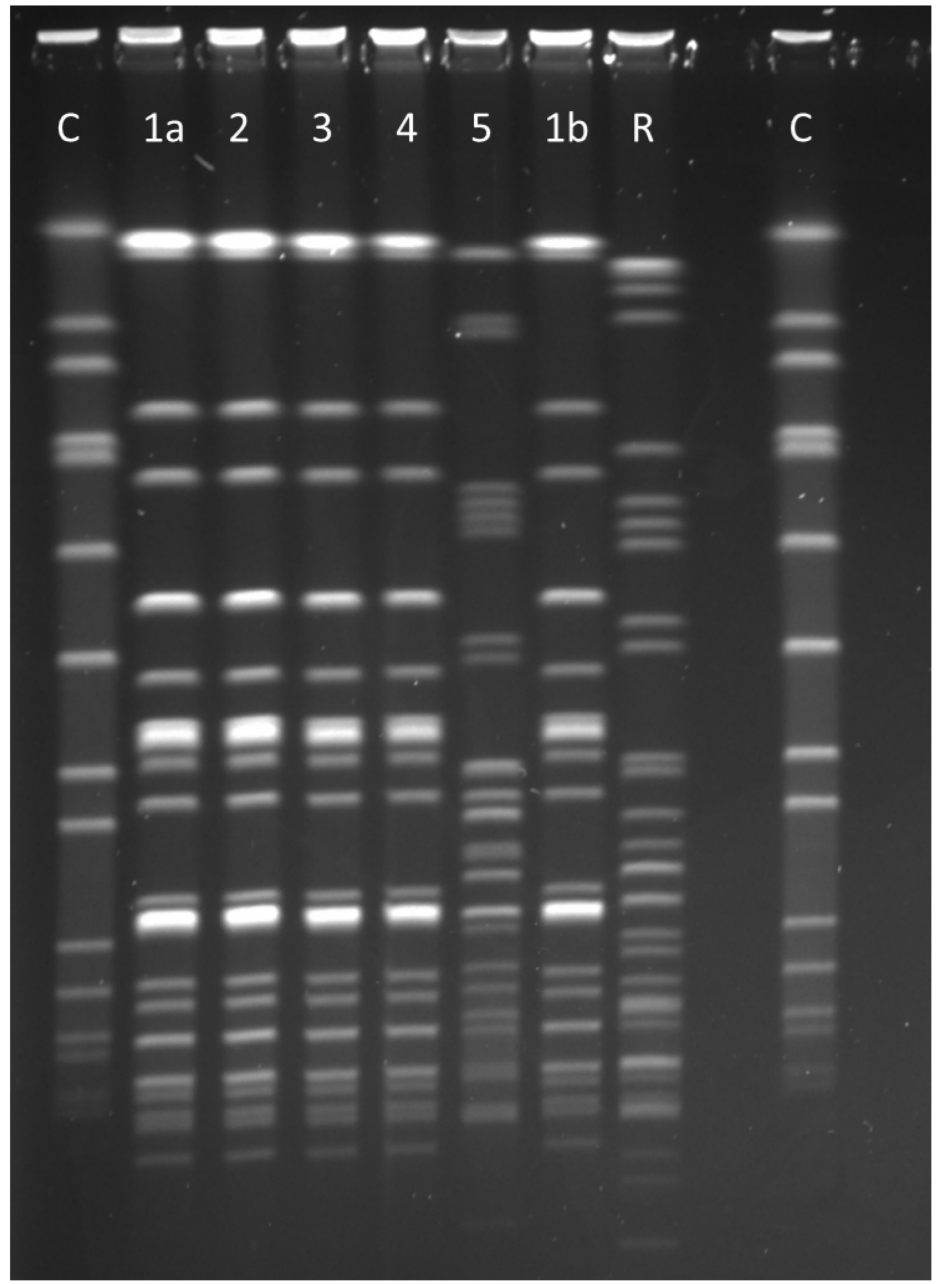
Pulsed-field gel electrophoresis C=St*aphylococcus aureus* quality control strain NCTC^®^ 8325; R=In-house *Serratia marcescens* reference strain; 1a, 2, 3 and 4=*Serratia marcescens* isolates (rectal specimens) of the four cluster patients showing the identical pulsotype (monoclonal pattern); 1b=*Serratia marcescens* blood culture isolate of patient 1 showing the same pattern as the rectal colonization isolate (1a); 5=*Serratia marcescens* isolate of a patient not belonging to the cluster.

The index patient developed an Sm BSI after initial rectal colonization. Empiric antibiotic therapy included meropenem and tobramycin. The patient recovered quickly with no foreseeable long-term effects to date. An initial PFGE was performed within two weeks that included the colonizing strains of all four patients. The isolates had an identical PFGE pattern. Figure 2 (Fig. 2) shows the result of a PFGE comprising the blood culture isolate of the first patient. 

In addition, breastmilk from the mother of the first patient was examined; however, Sm was not detected. No further cases occurred in the NICU with a turnover of 29 new admissions and 18 transfers by the time the remaining pair of twins with Sm were discharged from the NICU. All measures were continued until this discharge. However, the additional weekly screening targeting Sm was performed for another two weeks thereafter.

## Discussion

Implementation of this multimodal infection control response led to the immediate containment of the cluster. The response targeted various elements. Frequent and sensitive microbiological screening to quickly detect positive patients and monitor potentially ongoing transmission was essential. Repeated screening has also been described in other Sm clusters and is an important tool in outbreak control [2], [7]. To increase sensitivity, we made use of the intrinsic resistance of Sm against colistin. A similar approach using an enrichment broth containing colistin has been described [8]. In contrast to the use of an enrichment broth, the colistin disk allows a matrix-assisted laser desorption/ionization time-of-flight mass spectrometry-based identification of colonies growing in the inhibition zone after incubation for 18–24 h.

All stakeholders were involved to guarantee a prompt and continuous flow of information by implementing an interdisciplinary task force.

The following measures, which are often reported in outbreak literature, are specifically aimed at interrupting transmission:

1. spatial isolation (e.g., private room),
2. exclusively assigned staff for Sm positive patients,
3. strengthening hand hygiene, and
4. discharge of Sm carriers [1], [2], [3], [4].

Reinforcing hand hygiene appears to be of particular importance [5], [9], as contaminated hands are a relevant route of transmission [1]. Contaminated hands might have also played a role in this cluster, as the pairs of twins were each located in different rooms, but cared for by the same staff prior to Sm detection. 

Timely discharge and transfer of Sm positive patients is important to remove or minimize a potential source of transmission. It is noteworthy that one pair of twins stayed in the NICU for a total of 43 days. Due to this long period, transmission control was a challenge, considering all patient admissions and transfers.

Clinical alertness towards the development of infections, especially BSI, is essential to ensure early administration of adequate empiric antibiotic therapy for suspected invasive infection in Sm-colonized patients. Here, one of the four infants developed BSI, which is a known and feared phenomenon in Sm clusters [4]. Molecular characterization of bacterial isolates in a cluster is crucial for a better understanding of the transmission process. PFGE was used in this case. Sequencing-based methods are also emerging for Sm [5], [10]. One benefit of sequencing-based methods with a standardized nomenclature (e.g., core genome multilocus sequence typing) is that data generated from different settings (e.g., different outbreaks) can be compared quite easily.

Sm might persist in the environment; it was found in environmental samples from Sm clusters [1]. Therefore, environmental microbiological sampling should be performed – especially when the epidemiological pattern suggests a point source rather than patient-to-patient transmission. We had planned to examine inanimate surfaces (e.g., incubators, ultrasound equipment) and disinfectant solution in dispensers if more cases arose. 

In the present case, the frequency of regular cleaning was already quite high. However, intensified cleaning is reported as an outbreak control measure [2], [3]. We refrained from testing staff. Complete or partial closure of an NICU is an *ultima ratio* measure in Sm outbreaks [2]. However, it is highly disruptive and often not feasible. Fortunately, this was not necessary in our case. 

In conclusion, rapid implementation of a comprehensive and multimodal infection control response starting immediately with the first identified Sm patient is required to avoid a full-blown outbreak. Frequent and sensitive microbiological screening of all patients, rigorous spatial isolation of colonized infants and reinforcing adherence to hand hygiene are essential. De-escalation of measures is possible once the last Sm patient has been discharged.

## Notes

### Competing interests

The authors declare that they have no competing interests.

### Funding

This research did not receive any specific grant from funding agencies in the public, commercial, or non-profit sectors.

### Authors’ contributions

All authors contributed to the manuscript according to the ICMJE (International Committee of Medical Journal Editors) recommendations and were involved in data acquisition, analysis and interpretation. CB, PC, CBO and F-CB prepared the manuscript. CB organized the drafting process. BB, CP, CB, EE and F-CB supervised the infection control response on the ward. PC and DS were involved in microbiologic diagnostics. All authors critically revised the manuscript, account for accuracy and correctness and have read and agreed to the final draft before submission. CBO and PC are contributed equally as first author.
